# Presence of Multiple Mycotoxins and Other Fungal Metabolites in Native Grasses from a Wetland Ecosystem in Argentina Intended for Grazing Cattle

**DOI:** 10.3390/toxins7083309

**Published:** 2015-08-20

**Authors:** María J. Nichea, Sofia A. Palacios, Stella M. Chiacchiera, Michael Sulyok, Rudolf Krska, Sofia N. Chulze, Adriana M. Torres, María L. Ramirez

**Affiliations:** 1 Departamento de Microbiología e Inmunología, Facultad de Ciencias Exactas Fco-Qcas y Naturales, Universidad Nacional de Río Cuarto, Ruta 36 Km 601, Río Cuarto 5800, Córdoba, Argentina; E-Mails: mnichea@exa.unrc.edu.ar (M.J.N.); spalacios@exa.unrc.edu.ar (S.A.P.); schulze@exa.unrc.edu.ar (S.N.C.); atorres@exa.unrc.edu.ar (A.M.T.); 2 Departamento de Química, Facultad de Ciencias Exactas Fco-Qcas y Naturales, Universidad Nacional de Rio Cuarto, Ruta 36 Km 601, Río Cuarto 5800, Córdoba, Argentina; E-Mail: schiacchiera@exa.unrc.edu.ar; 3 Department IFA-Tulln, BOKU Vienna, Konrad Lorenzstr 20, Tulln A-3430, Austria; E-Mails: michael.sulyok@boku.ac.at (M.S.); rudolf.krska@boku.ac.at (R.K.)

**Keywords:** Poaceae, grasses, mycotoxins, *Fusarium*, cattle feed, bacterial and fungal metabolites

## Abstract

The aim of this study was to evaluate the occurrence of several fungal metabolites, including mycotoxins in natural grasses (Poaceae) intended for grazing cattle. A total number of 72 and 77 different metabolites were detected on 106 and 69 grass samples collected during 2011 and 2014, respectively. A total of 60 metabolites were found across both years. Among the few mycotoxins considered toxic for ruminants, no samples of natural grasses were contaminated with aflatoxins, ochratoxin A, ergot alkaloids, and gliotoxin, among others. However, we were able to detect important metabolites (toxic to ruminants) such as type A trichothecenes, mainly T-2 toxin and HT-2 toxin (up to 5000 µg/kg each), and zearalenone (up to 2000 µg/kg), all at very high frequencies and levels. Other fungal metabolites that were found to be prevalent were other *Fusarium* metabolites like beauvericin, equisetin and aurofusarin, metabolites produced by *Alternaria* spp., sterigmatocystin and its precursors and anthrachinone derivatives. It is important to point out that the profile of common metabolites was shared during both years of sampling, and also that the occurrence of important metabolites is not a sporadic event. Considering that this area of temperate grassland is used for grazing cattle all year long due to the richness in palatable grasses (Poaceae), the present work represents a starting point for further studies on the occurrence of multi-mycotoxins in natural grasses in order to have a complete picture of the extent of cattle exposure. Also, the present study shows that the presence of zeranol in urine of beef cattle may not be a consequence of illegal use of this banned substance, but the product of the natural occurrence of zearalenone and α-zearalenol in natural grasses intended for cattle feeding.

## 1. Introduction

Extensive beef production is a distinctive feature of Argentina. Beef production is generally based on grazing native grasses and cultivated pastures [1]. Nearly all the cattle are raised through grazing, with only 1.2% finished in corrals. Grazing throughout the year produces leaner beef cuts with less cholesterol and greater polyunsaturated fatty acids than beef finished on a high grain diet in feedlot [2]. Conventional cattle grazing in Argentina provides a desirable product for internal and external markets [3]. The prospects for Argentinean beef exports are promising, mainly due to the country’s recent advancements in sanitary status. Argentina has been declared free of foot and mouth disease with vaccination, and also has been assigned the best possible status for a low Bovine Spongiform Encephalopathy (BSE) risk. These achievements, together with the fertile soil and climate characteristics for beef production, are driving forces for the increase in the country’s beef exports [1].

Mycotoxins are naturally occurring compounds or secondary metabolites produced by fungi growing on plants in the field or during storage. Mycotoxins can contaminate raw agricultural products before and/or after harvest. Numerous mycotoxins can be produced by fungi invading plant material; however, only few mycotoxins have been recognized as toxic to ruminants. Mycotoxins relevant for ruminant’s health are aflatoxins, ochratoxin A, zearalenone, fumonisins (B_1_ and B_2_), trichothecenes, ergot alkaloids, and gliotoxin among others [4].

The negative effect of mycotoxins on the growth and health of livestock makes them a major problem for many production systems. Mycotoxicosis symptoms depend on the type of mycotoxin, the amount and duration of the exposure, the age, health and sex of the exposed individual, as well as on the dietary status and interactions among toxins. Low levels of mycotoxins may cause reduction decrease in food intake and performance, such as lowered milk production or decrease in body weight gain. Moderate levels of feed contamination frequently result in impaired resistance to infections, increased susceptibility to stress and reduced fertility. High levels of contamination may produce clinical disease, liver and kidney damage, oedema, increased blood clotting time and haemorrhaging, as well as altered digestion, absorption and metabolism of nutrients. Ruminants are less sensitive to the negative mycotoxin effects since rumen microbiota can effectively degrade and deactivate mycotoxins, hence protecting the animal [5,6].

Beef animals in Argentina are finished either solely on natural grasses or pastures, on pastures with supplemented feeding (in most cases corn or sorghum silage) or in feedlots [7]. In Argentina, several reports showed mycotoxin contamination in cattle feed ingredients such as corn grains, mixed rations (corn, protein concentrate, *etc.*), corn silage, soybean pellet, wheat bran, *etc.* all used in feedlot rearing practice [8]. However, there is little information on the natural occurrence of mycotoxins in natural grasses (uncultivated) devoted to cattle grazing.

Of particular concern to the Argentinean livestock industry is that zearalenone is chemically similar to the growth promoting α-zearalanol (zeranol), which is banned in Argentina as well as in the EU. In the last five years, zeranol has been detected in bovine urine during the routine analysis of beef cattle farms (enrolled as EU exporter) as part of a national residue control plan by the central governing authority, the National Service for Health and Food Quality (SENASA). It is important to remark that on those cattle farms, the cattle were raised through grazing of natural grasses, without any external inputs. The present research was primarily undertaken to ascertain if zearalenone was present in natural grasses on two beef cattle farms, in which a positive urine sample for zeranol from cattle had been previously found. It is well known that zeranol can be formed from α-zearalenol and zearalenone *in vivo* in cattle [9]. Several reports from New Zealand and Northern Ireland have shown that zeranol might occur naturally in urine and bile from sheep and cattle, following metabolism of the mycotoxins zearalenone and α-zearalenol that can contaminate animal feedstuffs [9,10]. Thus, the finding of zeranol in an animal might, on its own, be insufficient proof that malicious abuse of zeranol has occurred. As natural grasses (Poaceae) were the unique feed source for the cattle raising in the aforementioned cattle farming, we wanted to probe for the first time in Argentina that natural grasses were contaminated with zearalenone and that it was the possible source of zeranol. The two farms in this study were located at a Ramsar Wetland site (27°20'S 58°50'W, Ramsar site No. 1366) in Chaco province, Argentina [11]. This wetland ecosystem is one of the three most biodiverse biomes of Argentina and it covers part of the Parana and Paraguay rivers floodplain complex at the eastern border of Chaco Province. The landscape consists of complex open water, aquatic vegetation, grasslands and gallery forests. This temperate grassland is used for grazing cattle all year long due to the richness of palatable grasses (Poaceae).

Based on what has been mentioned above, the aims of this study were to evaluate the occurrence of several fungal metabolites including mycotoxins in natural grasses intended for cattle grazing and to determine the co-occurrence of toxins that may be relevant to impairing cattle growth and health.

## 2. Results

### 2.1. Performance of the Applied Analytical Method

The performance characteristics of the analytical method obtained from five spiked blank samples are presented in Table 1 (only those analytes that have later been detected in the survey are shown). The limits of detection (LOD) ranged between 0.03 (averufin and festuclavine) and 20 µg/kg (kojic acid). Spiking experiments using five individual samples at two concentration levels revealed that the apparent recoveries are in general lower than those obtained with other (grain-based) matrices [12,13].

**Table 1:** Performance characteristics of the analytical method for all analytes detected in the investigated grass samples.

Analyte	Apparent Recovery (%)	LOD ^a^ (µg/kg)
3-Nitropropionic acid	63.3 ± 9.5	0.40
Agroclavine	59.5 ± 5.1	0.08
Altenuene	157.8 ± 40.0	2.00
Alternariol	100.0 ± 9.5	0.50
Alternariol methyl ether	96.6 ± 8.8	0.05
Altertoxin-I	77.9 ± 6.5	0.80
Aspinolid B	75.5 ± 13.2	0.80
Asterric acid	140.0 ± 9.6	3.00
Aurofusarin	75.3 ± 4.7	4.00
Averantin	76.5 ± 0.9	0.04
Averufanin ^b^	n.d. ^d^	-
Averufin	54.50 ± 2.50	0.03
Beauvericin	41.8 ± 6.0	0.04
Brefeldin A	66.5 ± 6.2	2.00
Brevianamid F	76.2 ± 2.1	0.40
Chanoclavin	73.5 ± 31.2	0.05
Chrysophanol	114.8 ± 11.8	1.50
Clonostachydiol	80.0 ± 21.0	0.80
Curvularin	127.6 ± 15.8	0.40
Cytochalasin B	66.8 ± 7.1	1.50
Cytochalasin C	66.8 ± 6.1	0.40
Cytochalasin D	62.5 ± 4.1	0.20
Cytochalasin H	57.2 ± 5.6	15.00
Cytochalasin J	59.2 ± 7.9	2.00
Dechlorogriseofulvin	73.7 ± 9.1	1.50
Diacetoxyscirpenol	61.0 ± 9.8	0.25
Dihydrogriseofulvin	69.7 ± 8.8	1.00
Emodin	131.3 ± 28.2	0.08
Enniatin B	77.7 ± 42.9	0.01
Equisetin	215.0 ± 38.7	0.08
Festuclavine	73.1 ± 17.3	0.03
Fumonisin B_1_	59.8 ± 8.1	3.00
Griseofulvin	69.7 ± 4.8	0.80
HT-2 toxin	68.3 ± 1.0	4.00
Kojic acid	83.4 ± 12.2	20.00
Macrosporin	125.8 ± 5.2	0.20
Moniliformin	113.7 ± 6.7	1.00
Monocerin	73.8 ± 3.0	0.40
Neosolaniol	79.2 ± 3.5	0.50
Nidurufin ^b^	n.d.	-
Nivalenol	41.3 ± 12.0	0.80
Norsolorinic acid	47.0 ± 15.1	0.40
Penicillide	110.9 ± 16.3	1.50
Physcion	114.3 ± 16.6	8.00
Pseurotin A	73.7 ± 48.7	0.50
Radicicol	117.0 ± 12.0	0.40
Secalonic acid D	79.1 ± 8.6	4.00
Skyrin	76.1 ± 4.6	0.20
Sterigmatocystin	69.6 ± 4.0	0.30
Sulochrin	89.7 ± 14.9	1.50
T-2 toxin	64.1 ± 3.6	0.80
Tentoxin	56.2 ± 11.9	0.20
Tenuazonic acid	407.4 ± 108.9	3.00
Tryptophol	79.7 ± 6.4	15.0
Versicolorin A	103.7 ± 3.6	0.40
Versicolorin C ^c^	n.d.	-
Zearalenone	124.6 ± 10.9	0.30
Zearalenone-4-sulfate	143.1 ± 16.1	0.40
α-zearalenol	120.6 ± 17.2	0.80
β-zearalenol	109.3 ± 15.6	0.80

^a^ LOD, limit of detection; ^b^ No standard available, estimation of concentration based on response and recovery of averufin; ^c^ No standard available, estimation of concentration based on response and recovery of versicolorin A; ^d^ n.d.: not determined.

### 2.2. Occurrence of Fungal Metabolites in Natural Grass Samples

**Figure 1:**
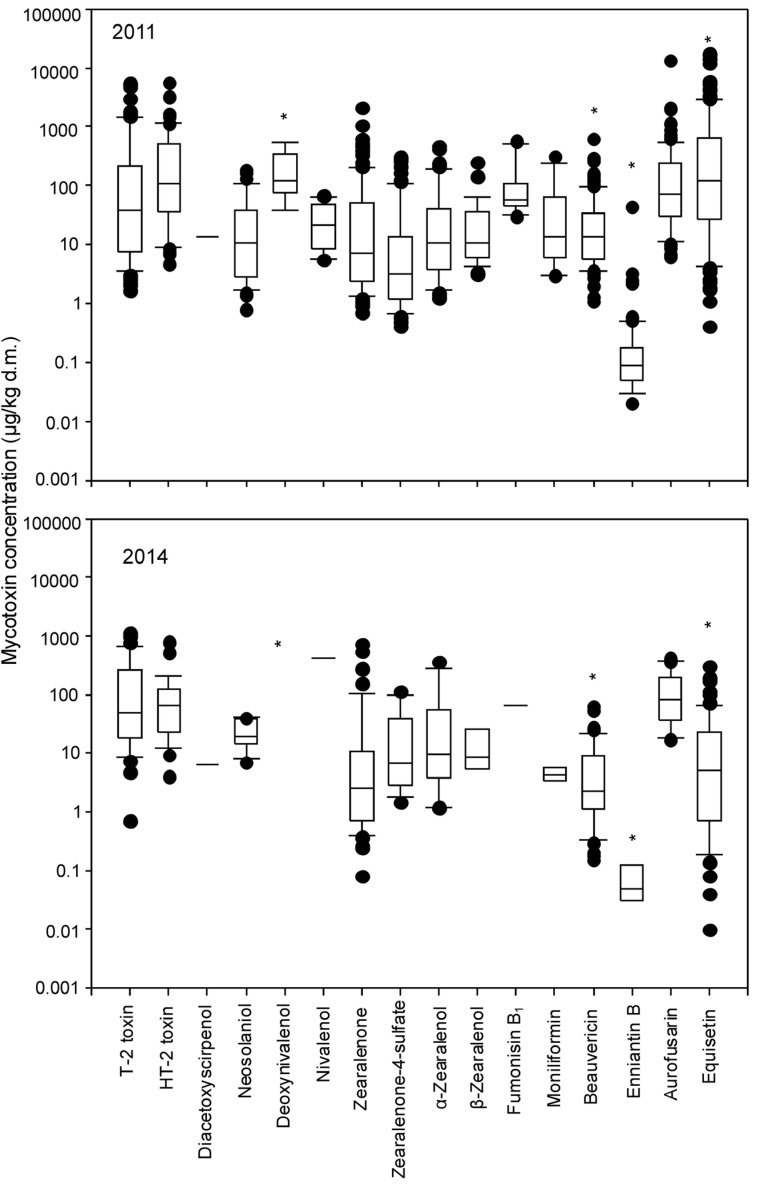
Box-plot for *Fusarium* mycotoxin concentration in grass samples during both years of study. (* for a particular toxin denotes significant differences between both years evaluated according to Tukey Test (*p* < 0.001)).

A total number of 72 and 77 different metabolites were detected on grass samples collected during 2011 and 2014 years, respectively. A total of 60 metabolites were shared on both years evaluated. Data on shared mycotoxin prevalence between both sampling years as well as related median and maximum concentration in the positive samples are compiled in Table 2. The list of the most prevalent metabolites was similar between both years; despite this, there was a lower prevalence/or lower concentration in the grass samples from 2014. It was noticeable that, all the non-shared metabolites found in a particular year were detected in very few samples, these being unimportant metabolites in terms of toxicity for ruminants (Data not shown). The only exception was the presence of deoxynivalenol in grass samples collected during 2011. As an example, Figure 1 shows contamination levels of 16 *Fusarium* mycotoxins detected in both years of sampling (except deoxynivalenol), and significant differences (*p* < 0.05) were found in the levels between both years in deoxynivelenol, beauvericin, enniantin B and equisetin, which appeared to be lower during 2014 in comparison with those collected during 2011.

In respect to *Fusarium* mycotoxins present in natural grass samples during both years evaluated, it was observed that during 2011, beauvericin and equisetin were present in all the samples and monocerin, zearalenone and aurofusariun were present in ≥90% of the samples. Other *Fusarium* mycotoxins detected at a prevalence of 40%–70% were HT-2 toxin, α-zearalenol, zearalenone-4 sulfate, T-2 toxin and enniantin B. Neosolaniol, β-zearalenol, nivalenol, moniliformin and fumonisin B_1_ were detected at frequencies of 36%, 28%, 15%, 12% and 11%, respectively. Deoxynivalenol, enniantin A and A_1_, culmorin and diacetoxyscirpenol were found in low abundance ≤10%.

**Table 2:** Occurrence and concentrations of the positive identified analytes in natural grass samples during 2011 and 2014.

Analyte	N° of Positives	Concentration of Positive Samples from 2011 (µg/kg d.m.)				N° of Positives	Concentration in Positive Samples from 2014 (µg/kg d.m.)			
		Median	75 Percentile	90 Percentile	Max		Median	75 Percentile	90 Percentile	Max
T-2 toxin	67/106	37.8	218	1375	5438	39/69	47.2	243	559	1095
HT-2 toxin	52/106	110	474	1149	5651	29/60	66.5	117	205	783
Diacetoxyscirpenol	2/106	13.6	21.5	21.5	21.5	2/69	6.49	10.5	10.5	10.5
Neosolaniol	36/106	10.4	39.0	94.5	187	11/69	19.4	38.1	39.6	39.9
Nivalenol	15/106	21.0	44.3	63.6	66.9	2/69	422	832	832	831
Zearalenone	95/106	7.20	50.3	203	2120	56/69	2.50	10.0	80.2	712
Zearalenone-4-sulfate	67/106	3.10	13.0	99.4	303	17/69	6.81	35.7	87.4	111
α-zearalenol	53/106	10.7	39.4	181	464	12/69	9.37	45.1	154	360
β-zearalenol	28/106	10.9	34.9	53.9	245	7/69	8.35	24.2	47.5	52.8
Fumonisin B_1_	11/106	57.2	97.0	442	566	2/69	64.0	98.7	98.7	98.7
Moniliformin	12/106	13.5	56.2	152	313	3/69	4.38	5.3	5.64	5.64
Beauvericin	106/106	13.5	33.2	95.1	624	43/69	2.23	9.1	18.5	63.4
Enniatin B	74/106	0.09	0.17	0.47	43.2	4/69	0.05	0.10	0.14	0.14
Aurofusarin	96/106	71.4	231	508	13238	25/69	80.7	195	365	409
Equisetin	106/106	118	624	2841	17264	63/69	2.21	12.8	54.8	297
Sterigmatocystin	96/106	4.15	16.5	42.6	733	41/69	6.78	15.1	53.8	147
Averantin	85/106	1.60	5.07	19.4	499	65/69	1.16	2.60	25.3	319
Averufanin	51/106	0.70	1.55	4.44	17.5	21/69	0.20	0.54	3.73	4.65
Averufin	67/106	3.80	8.57	20.4	173	67/69	2.78	8.00	49.3	401
Versicolorin A	20/106	0.65	1.35	27.4	46.5	38/69	3.08	11.9	103	719
Versicolorin C	33/106	1.50	2.42	4.22	25.3	47/69	3.36	10.1	43.9	209
Nidurufin	41/106	0.60	41.2	91.1	228	30/69	0.87	3.58	10.1	28.3
Norsolorinic acid	59/106	3.60	23.4	93.6	257	44/69	3.55	6.76	23.7	63.1
Kojic acid	39/106	206	329	404	522	66/69	103	127	139	187
3-nitropropionic acid	32/106	2.70	5.70	9.94	28.8	37/69	3.77	1.91	28.0	102
Aspinolid B	40/106	39.2	82.6	186	602	27/69	10.0	25.0	43.7	423
Asterric acid	44/106	33.6	58.8	141	346	26/69	22.9	58.3	140.2	223
Sulochrin	42/106	23.7	62.6	90.1	198	30/69	18.3	52.7	283	172
Pseurotin A	31/106	4.70	8.37	13.4	15.2	10/69	7.88	23.4	148	29.5
Agroclavine	2/106	11.2	14.0	14.0	14.0	1/69				
Chanoclavin	69/106	2.60	49.1	180.6	2259	31/69	2.44	45.1	439	815
Festuclavine	5/106	32.1	93.6	98.4	98.4	5/69	7.63	75.6	215	215
Secalonic acid	28/106	72.9	116	229	1431	7/69	36.5	202	230	236
Griseofulvin	30/106	18.9	150	594	5880	4/69	23.4	75.0	123	123
Dechlorogriseofulvin	18/106	16.2	124	270	3904	2/69	20.0	32.5	32.5	32.5
Dihydrogriseofulvin	20/106	17.4	119	333	5720	3/69	14.2	55.6	69.4	69.4
Curvularin	73/106	9.30	39.7	358	5362	14/69	5.10	8.62	121	934
Brefeldin A	2/106	1648	2988	2988	2988	2/69	745	874	874	875
Penicillide	7/106	5.70	10.1	14.1	14.9	1/69	15.4			
Tenuazonic acid	28/106	31.4	59.4	141	222	27/69	2.43	4.67	11.3	178
Alternariol	105/106	65.3	160.	349	1036	61/69	17.4	58.0	136	1021
Alternariol methyl ether	67/106	12.6	33.3	69.2	377	67/69	10.5	20.2	39.2	160
Tentoxin	90/106	1.60	4.30	12.8	324	21/69	3.76	21.3	136	252
Altenuene	23/106	10.3	15.5	19.8	28.4	2/69	14.3	18.7	18.7	18.7
Altertoxin-I	75/106	5.00	9.40	15.6	383	5/69	1.43	1.86	2.72	2.72
Macrosporin	60/106	2.85	8.90	21.8	50.4	17/69	1.95	4.53	19.2	76.2
Clonostachydiol	13/106	11.5	21.4	53.0	121	3/69	9.97	71.9	92.6	92.6
Cytochalasin B	10/106	90.0	136	925	1616	6/69	32.6	42.6	91.6	97.0
Cytochalasin C	14/106	27.9	64.6	264	412	3/69	33.6	38.1	39.5	39.5
Cytochalasin D	10/106	24.6	67.2	87.6	106	6/69	9.28	10.3	24.1	25.6
Cytochalasin H	9/106	368	1152	1802	2003	4/69	163	638	1064	1064
Cytochalasin J	20/106	40.8	133	215	434	4/69	122	436	677	677
Monocerin	106/106	66.7	215	1916	6745	65/69	20.7	46.9	642	7289
Brevianamid F	33/106	8.00	11.2	17.1	34.3	18/69	32.3	36.1	49.0	62.5
Tryptophol	75/106	73.1	113	217	466	67/69	67.3	226.8	1070	2513
Emodin	106/106	177	471	1551	3725	67/69	62.1	95.4	282	5401
Chrysophanol	104/106	41.1	77.8	142	15952	63/69	15.5	24.8	47.6	120
Physcion	74/106	69.9	166	391	17752	7/69	31.7	35.4	45.9	48.4
Skyrin	105/106	5.90	14.2	38.3	241	55/69	1.93	2.67	5.42	13.7
Radicicol	50/106	33.2	95.0	182	380	20/69	17.5	56.5	75.0	210

During 2014, monocerin and equisetin were also present in ≥90% whereas zearalenone was present in 81% of the samples analyzed. Other *Fusarium* mycotoxins detected at a prevalence of 40%–70% were T-2 toxin, HT-2 toxin and beauvericin. Aurofusarin, zearalenone-4 sulfate, α-zearalenol and neosolaniol were detected at frequencies of 36%, 25%, 17% and 16%, respectively. β-zearalenol, enniantin B, moniliformin, diacetoxyscirpenol, nivalenol and fumonisin B_1_ were found in low abundance ≤10%.

In particular, out of 106 natural grass samples collected during 2011, 95 were contaminated with zearalenone in concentrations ranging from 0.7–2120 µg/kg d.m. (mean = 84.5 µg/kg). Also, 52 grass samples were co-contaminated with zearalenone and α-zearalenol, both precursors of zeranol, while 22 samples showed co-occurrence of zearalenone, α-zearalenol and β-zearalenol. In the 2014 grass samples, 56 out of 69 samples contaminated with zearalenone in concentrations ranging from 0.3–711.80 µg/kg d.m. (mean = 41.40 µg/kg d.m.) were found. Also, seven grass samples were co-contaminated with zearalenone and α-zearalenol, both precursors of zeranol, and seven samples showed co-occurrence of zearalenone, α-zearalenol and β-zearalenol.

Alternariol was the most frequent (99%) *Alternaria* mycotoxin found on natural grasses during 2011. Tentoxin, altertoxin-1, alternariol monomethyl ether and macrosporin were detected in frequencies ranging from 85% to 57%. Tenuazonic acid was detected in 26% of the samples analysed.

Alternariol monomethyl ether was the most frequent (97%) *Alternaria* mycotoxin found on natural grasses during 2014. Alternariol, tenuazonic acid, tentoxin and macrosporin were detected in frequencies ranging from 88% to 25%. Altertoxin-I was detected at very low frequency (7%) in the samples analysed.

Aflatoxin was not detected in any grass sample analysed during both years evaluated, but sterigmatocystin and some of its precursors exhibited a very high prevalence (sterigmatocystin: 90% in 2011 and 60% in 2014; averantin; 80% in 2011 and 99% in 2014). Averufin, norsolorinic acid, averufanin, versicolorin C, nidurufin and versicolorin A were detected in frequencies >20% during both years.

Chanoclavin and curvularin were the most frequent *Penicillium* mycotoxin found on natural grass during both those years analyzed. Another eight metabolites (agroclavine, festuclavine, secalonic acid, dihydrogriseofulvin, dechlorogriseofulvin, brefeldin A and penicillide) produced by *Penicillium* species were also detected, but in low abundance ≤27% (see Table 1). Although some metabolites such as griseofulvin, dihydrogriseofulvin, dechlorogriseofulvin among others were found in low frequency, the maximum concentrations detected were very high in some samples.

Anthraquinones such as chrysophanol, emodin and skyrin that may be produced both by fungi and plants, were detected in high frequency (>80%) during both years and also at very high levels in some samples. However, physcion was detected in high frequency just in 2011 samples.

Cytochalasin B, C, D, H and J were detected in low frequency (≤20%), but some of them such as cytochalasin B was found in high concentration in some samples (1616 µg/kg).

The bacterial metabolite monactin was found at low prevalence (≤10%) and also at low levels only in grass samples collected during 2011 (data not shown).

## 3. Discussion

To our knowledge, the present study represents the first survey on multi-mycotoxin contamination occurrence in natural grasses used for beef animal production. It was noticeable that the LC-MS/MS method applied possessed the performance characteristics required to obtain accurate results. Out of the 175 natural grass samples analysed, all showed contamination, being co-occurrence the rule. The use of the multi-metabolites LC-MS/MS method allowed us to ascertain the extent of the natural grasses’ contamination. It is important to remark that the beef cattle grazing on both farms evaluated during 2011 and 2014 did not show any symptoms of mycotoxicosis. Since our main goal was to demonstrate that the farmers did not abuse the use of zeranol, we obtained samples in 2011, a year after the finding of zeranol in urine among cattle from both farms, in the paddocks where the cattle were grazing the year before. As we used a multi-toxin analytical method, we were able to detect co-occurrence of zearalenone and several metabolites for the first time in natural grasses. We conducted other sampling during 2014 in order to probe that our first findings were not a sporadic event, and to probe that it is normal to find zearalenone and other mycotoxins on natural grasses (uncultivated).

Numerous mycotoxins can be produced by fungi invading plant material; however, only few mycotoxins have been recognized as toxic to ruminants. The toxins detected in the present survey included the major mycotoxins of concern for ruminant health such as zearalenone, and trichothecenes, and others still not evaluated [4].

Common knowledge on animal mycotoxicosis indicates that ruminant animals are among the least susceptible animal species, as the rumen microflora effectively degrade and inactivate mycotoxins, hence protecting the animal [5]. Some microbes from the rumen have been identified for their ability to degrade mycotoxins or plant toxins. Among the first mycotoxins shown to be detoxified by ruminants were ochratoxin A [14], and aflatoxin B_1_ [15,16]. The metabolism of different mycotoxins potentially encountered by ruminants has also been investigated, and it has been found that the mycotoxins zearalenone, T-2 toxin, diacetoxyscirpenol and deoxynivalenol were well metabolized by whole rumen fluid, whereas aflatoxin B_1_ and ochratoxin A were not [17]. Kennedy *et al.* [9] reported that 90% of zearalenone was hydrolyzed to α-zearalenol by rumen microbes. Although the α form of zearalenone is more estrogenic than its parent form, due to the low rate of absorption, ruminants are less susceptible to zearalenone toxicity [18].

Among the few mycotoxins considered toxic for ruminants, none of the natural grass samples collected in both years were contaminated with aflatoxins, ochratoxin A, ergot alkaloids, gliotoxin among others. However, we were able to detect important metabolites such as trichothecenes type A, and B, sterigmatocistin and zearalenone.

Data on zearalenone and derivates detected during the present study are of concern if we consider, as an example, that beef cattle need to eat 10% of their weight body per day; an animal of 400 kg needs to eat 40 kg of natural grasses a day. Considering our results, 40 kg of natural grasses will result in an average daily intake of 3.38 mg of zearalenone and 2.0 mg α-zearalenol per animal. Kleinova *et al.* [19] when feeding heifers with similar amounts of zearalenone (oat contaminated with 2.74 mg zearalenone per animal) and a control group with zeranol implant (25 mg), found in urine samples of the treated and control animals similar concentrations of zeranol (α-zearalanol) and taleranol (β-zearalanol). In addition, in the heifers that have consumed oat contaminated with zearalenone, zearalenone, α-zearalenol and β-zearalenol in urine were also found. The present study showed that the presence of zeranol in urine of beef cattle could be not a consequence of illegal use of this banned substance, but the product of the natural occurrence of zearalenone and α-zearalenol in natural grasses intended for cattle feeding. The high prevalence of these metabolites during both years is remarkable being that their presence is not a sporadic event.

It is important to highlight the presence of zearalenone-4-sulfate in natural grass samples during both years. Sulfo-conjugation is part of the phase II detoxification process that plants and animals use to inactivate mycotoxins and other xenobiotics. It has also been demonstrated by Berthiller *et al.* [20] that *Arabidopsis thaliana* seedlings treated with zearalenone produced also zearalenone-4-sulfate. Zearalenone-4-sulfate formation seems to be a mechanism of self-protection. Despite its chemical alteration, there is evidence that the above mentioned metabolite has a similar toxic potential to those of their precursors when ingested with food, as attached functional groups like sulfate residues are likely to be enzymatically cleaved during digestion [21].

Among the type A trichothecenes, T-2 toxin, HT-2 toxin, neosolaniol and diacetoxyscirpenol were found in 2011 and 2014 and we did not find any significant differences in this group of mycotoxins between both years analysed. Some grass samples from 2011 showed very high concentration of T-2 toxin and HT-2 toxin, up to 5000 µg/kg d.m. each during 2011. T-2 and HT-2 toxin, the most prominent type A trichothecenes, often found together in plants, are some of the most toxic trichothecene detected in feed for cattle. Ruminants can rapidly de-acetylate T-2 toxin to HT-2 [22]. It is often difficult to distinguish the effects of T-2 toxin from HT-2 toxin *in vivo*; therefore, it is reasonable to sum up the concentrations of these toxins to evaluate clinical effects. T-2 toxin ingestion results in a severe irritation of the upper digestive tract, including a hemorrhagic ruminitis, due to its cytotoxic effects. The T-2 toxin is also believed to induce immune-suppression in cattle by decreasing serum concentrations of IgM, IgG and IgA, neutrophil functions and lymphocyte blastogenesis. Bovine infertility and abortion in the final trimester of gestation have also resulted from the consumption of feed contaminated with T-2 [6]. With the exception of T-2 toxin, cattle have not been adversely affected by others trichothecenes. The high levels of type A trichothecenes (especially T-2 and HT-2 toxins) can be explained due to the *Fusarium* species contamination. This assertion is supported by the mycological analysis of 2011 grass samples, which revealed that 100% of the samples were contaminated with *Fusarium*, being *F. armeniacum* the most common species found. Also, we have demonstrated that 50 selected *F. armeniacum* isolates were able to produce a broad range of type A trichothecenes (including T-2, HT-2, neosolaniol among others) [23]. Consequently, *F. armeniacum* could be responsible for the high prevalence of type A trichotheces on natural grasses analysed during the present study.

Deoxynivalenol and nivalenol were the only trichothecene type B found in natural grasses, at very low frequencies and levels during 2011. Deoxynivalenol was not present in 2014, while nivalenol was found at very low frequency but at higher concentrations than in 2011. Deoxynivalenol, the most prominent type B trichothecene present in cattle feed worldwide, is not considered to be acutely toxic; it is considered to be a major cause of economic loss due to reduced performance. Clinical signs include gastrointestinal problems, soft stools, diarrhea, increased susceptibility to other diseases and decreased performance. Cattle are resistant to the emetic effects of deoxynivalenol, but reduced food intake was observed at 10–20 mg/kg in ruminants [24]. During the present work, deoxynivalenol was detected in few samples (*n* = 9) and at concentrations lower than the guidance value provided by the European Commission for bovine feed. Nivalenol was found in low prevalence (22% and 3%) with all sample values below 831.80 µg/kg d.m., thereby presenting contamination levels within the range of surveys conducted elsewhere [25,26,27,28,29]. The risk associated with chronic exposure to low levels of nivalenol in animal feed is difficult to evaluate due to the limited data available in farm animals.

It is generally assumed that within the rumen the protozoal population has the highest capacity to detoxify ingested myctoxins, but this may vary between different classes of mycotoxins. The contribution of bacteria and other rumen organisms might have been underestimated, as generally only the overall capacity to degrade a given mycotoxin has been tested [27]. Several mycotoxins are, however, able to modify the rumen microbiota as they exert antimicrobial, anti-protozoal and antifungal activity; typical examples are patulin, fusaric acid, beauvericin and enniantins. The findings that mycotoxins impair the rumen microbiota correspond to the observation in clinical practice where, following a period of feeding mould contaminated silage to dairy cows, a reduced filling of the rumen, poor feed conversion, and mild diarrhea are observed [30,31]. The co-occurrence of mycotoxins observed in the present study is relevant considering their potential effects on the rumen microbiota.

Enniatins and beauvericin are cytotoxic cyclic hexadepsipeptides produced by several *Fusarium* species and are known to be toxic to insects, bacteria and fungi [32]. Enniatin B was the most prevalent enniatin mycotoxin, present in 70% of the samples during 2011, but all the enniatins were found in very low concentrations. In 2014 samples, enniantin B was the only one enniantin detected at very low frequency and levels. Beauvericin was present in all analysed grass samples in 2011 and in 43 during 2014, at levels within the concentration range reported by other studies [33,34]. There are no data that indicate that beauvericin has potential toxicity to cattle.

The occurrence of alternariol, alternariol methyl ether, tentoxin and altertoxin I was high in the analysed samples (99%–70%) during 2011. Although, in samples from 2014, alternariol and alternariol methyl ether were present at very high frequencies (88% and 97%), tentoxin and altertoxin I were found at lower frequencies than in 2011. Overall, maximum levels of alternariol were higher than those reported in the literature with the exception of sunflower. *Alternaria* species produce more than 70 phytotoxins, but a small proportion of them has been chemically characterized and reported to act as mycotoxins to humans and animals. Some toxins such as alternariol, alternariol methyl ether, tenuazonic acid and altertoxins are described to induce harmful effects in animals, including fetotoxic and teratogenic effects. At present, knowledge of the possible effects of *Alternaria* toxins on farm and companion animals as well as the database describing the occurrence of these mycotoxins in feedstuffs are scarce and insufficient to assess the risk regarding *Alternaria* toxins for animal health [35].

Occurrence data on equisetin and monocerin on animal feed are very limited [36]. Both mentioned metabolites were found at very high frequencies in both years (>90%) and at very high concentration in some samples analysed in the present study. Equisetin was reported as a metabolite of *F. equiseti* and *F. semitectum* with weak activity against gram-positive bacteria and other cytotoxic activities [37]. Equisetin has been found as a natural contaminant in corn, wheat silage and corn silage but at concentrations lower than those obtained during the present study [36,38]. Monocerin is a polyketide fungal metabolite that exhibits antifungal, insecticidal, and plant pathogenic properties. It has been isolated from several fungal species [39]. Monocerin was present in all grass samples under study, at relatively high concentrations comparable with those found by Shimshoni *et al.* [36] on corn silage. The toxic effect of this metabolite on ruminants is still unknown.

Aflatoxins were not detected during the present study, although we were able to detect several precursors (some at very high frequencies) in the biosynthetic aflatoxin (AF) pathway. This suggests *Aspergillus versicolor* as the fungal producer The general accepted AF biosynthetic pathway scheme is: A hexanol CoA precursor → norsolorinic acid, NOR → averantin, AVN → hydroxyaverantin, HAVN → Oxoaverantin, OAVN → averufin, AVF → Hydroxyversicolorone, HVN → versiconal hemicetal acetate, VHA → versiconal, VAL → versicolorin B, VERB → versicolorin A, VERA → demethyl-sterigmatocystin, DMST → sterigmatocystin , ST O-methylsterigmatocystin, OMST → aflatoxin B_1_, AFB_1_ and aflatoxin G_1_, AFG_1_. After the VHA step, there is a branch point in the pathway that leads to AFB_1_ and AFG_1_ formation as well as AFB_2_ and AFG_2_ [40]. In particular ST, the penultimate precursor of AF, is produced by more than 50 fungal species, including *Aspergillus flavus*, *A. parasiticus*, *A. versicolor* and *A. nidulans*, of which *A. versicolor* is the most common source. ST shares its biosynthetic pathway with aflatoxins. *A. nidulans* and *A. versicolor* are apparently unable to biotransform ST into OMST, the direct precursor of AFB_1_ and AFG_1_. Consequently, substrates colonized by these fungi can contain high amounts of ST, while substrates invaded by *A. flavus* and *A. parasiticus* contain only low amounts of ST as most of it is converted into AFs. ST was detected in 90% and 60% of the grass samples analysed during 2011 and 2014, respectively. Owing to the structural similarities, AFs and ST share prominent toxic effects, including genotoxicity and carcinogenicity, being the AFs considered 150–200 times more potent than ST [41]. However, in contrast to AFs, only limited information on occurrence and toxicity of ST is available. Only limited data are available for other ruminants, but a case report describes haemorrhages and bloody diarrhea in cattle following exposure to ST [42]. Some grass samples showed very high levels of contamination with ST (up to 730 µg/kg d.m.) if we consider the guidance of the European Parliament [43] for feed, the concentration of ST must be regarded as significant.

Among the *Penicillium* metabolites, we were unable to detect in any samples patulin, mycophenolic acid, roquefortine C and PR toxin that are common mycotoxins found in grass silage around the world [44].

Several infrequently reported anthraquinone derivates produced by fungi and plants were found during the present research, such as emodin, its methy-derivate physcion and chrysophanol and its respective dimer skyrin. The high maximum concentration detected of the above mentioned metabolites is also remarkable, mainly in samples from 2011. All these anthraquinones are commonly found on plants belonging to the Poligonaceae family, and have been reported as antibacterial, anti-inflammatory, antiviral anti-ulcerogenic and anticancer agents. Also, all these compounds play well-documented roles as chemopreventive effects [45].

During this study we have detected one bacterial metabolite with antibiotic activity: monactin at very low frequency (<10%) and concentrations in 2011 grass samples. This metabolite is a member of the macrotetrolide complex produced by a range of *Streptomyces* species [46]. At present, no data are available regarding the effect of this antibiotic present in grasses on ruminants.

We did not detect any of the indole-diterpenoid alkaloids in the natural grasses under study. These compounds are produced by species of the *Claviceps* genus within the Hypocreales that can infest plant species belonging to Poaceae (family of the true grasses). In Argentina, mycotoxicosis among cattle has been reported due to the presence of indol-diterpenoid tremorgens, produced by *Claviceps paspali* present in grasses such as *Paspalum dilatatum* and *P. notatum* and by *Claviceps cynodontis* present on *Cynodon dactylon*. Also, ryegrass staggers caused by the endophytic fungi *Neotyphodium lolii* (that produce indol-diterpenoid alkaloids) in *Lolium perenne* occurs frequently in cattle in Argentina. Gangrenous ergotism caused by *C. purpurea* and *Festuca elation* has also been observed [47].

This study showed new and original data on the presence of multi-fungal and bacterial metabolites on natural grasses (non-cultivated) used for grazing cattle. Only few studies have investigated the presence of mycotoxins but just on cultivated grasses used as forage (mainly silage) for cattle feeding, most of them focus on few mycotoxins such as deoxynivalenol, zearalenone, fumonisins and aflatoxins [48,49,50].

## 4. Experimental Section

### 4.1. Sampling

Natural grass samples were obtained from two beef cattle farms located in the Chaco province of Argentina, included in the Ramsar site. One hundred and six grass samples (53 from each farm) were collected during July 2011, and 69 during July 2014. On each farm, a paddock of around 1000 ha was chosen for sampling. Each sample, corresponding to one plant, was cut at ground level and transported to the laboratory in a paper bag. The aerial harvested portions of plants, including leaf and stems, were immediately oven dried at 60 °C for 48 h or until constant weight, indicating that the entire aqueous portion was extracted from plant tissues. Since the sampling was done during winter, it was not possible to identify the grasses up to species level due to the absence of inflorescence but we can confirm that all belong to the Poaceae family.

The two cattle farms were located in the wetland of Chaco region in Argentina. This region covers part of the Parana and Paraguay rivers floodplain complex in the eastern border of Chaco Province and it is limited to the north by the Bermejo River which surrounds the city of Resistencia. The hydrological regimes of each river give rise to different pulses of flood and drought in these wetlands, regulating flood downstream and retaining waters in times of drought. The landscape is complex open water, aquatic vegetation, grasslands and gallery forests. The annual temperature ranges between 20 and 24 °C. Maximum absolute temperatures can peak at 46.5 °C. Mean annual rainfall is 1300 mm, concentrated in spring and summer. The most common species of grasses in the area under study are *Leersia hexandra*, *Luziola peruviana*, *Sorghastrum setosum*, *Spartina argentinensis*, *Cynodon dactylon* between others [51].

### 4.2. Mycotoxin Analysis

#### 4.2.1. Chemicals and Reagents

Methanol (LC gradient grade) and glacial acetic acid (p.a.) were purchased from Merck (Darmstadt, Germany), acetonitrile (LC gradient grade) from VWR (Leuven, Belgium), and ammonium acetate (MS grade) from Sigma-Aldrich (Vienna, Austria). Water was purified successively by reverse osmosis and an Elga Purelab ultra analytic system from Veolia Water (Bucks, UK) to 18.2 MΩ.

Standards of fungal and bacterial metabolites were obtained either as gifts from various research groups or from the following commercial sources: Romer Labs^®^ Inc. (Tulln, Austria), Sigma-Aldrich (Vienna, Austria), Iris Biotech GmbH (Marktredwitz, Germany), Axxora Europe (Lausanne, Switzerland) and LGC Promochem GmbH (Wesel, Germany). Stock solutions of each analyte were prepared by dissolving the solid substance in acetonitrile (preferably), acetonitrile/water 1:1 (*v*/*v*), methanol, methanol/water 1:1 (*v*/*v*) or water. Thirty-four combined working solutions were prepared by mixing the stock solutions of the corresponding analytes for easier handling, and were stored at −20 °C. The final working solution was freshly prepared prior to spiking experiments by mixing the combined working solutions.

#### 4.2.2. Extraction and Estimation of Apparent Recoveries

Three grams of each grass sample, previously pulverized in a mill with a 1 mm^2^ mesh (Cyclotech, Foss Tecator, Höganäs, Sweden), were weighed into a 50 mL polypropylene tube (Sarstedt, Nümbrecht, Germany) and 20 mL of the extraction solvent (acetonitrile/water/acetic acid 79:20:1, *v*/*v*/*v*) were added. For spiking experiments, 0.25 g sample was used for extraction. Samples were extracted for 90 min on a GFL 3017 rotary shaker (GFL, Burgwedel, Germany) and diluted 1 + 1 with dilution solvent (acetonitrile/water/acetic acid 20:79:1, *v*/*v*/*v*). Five microliters of the diluted extracts were subsequently injected [13].

#### 4.2.3. LC-MS/MS Parameters

The LC-MS/MS has been previously described by Vishwanath *et al.* [52], but has been transformed to a more sensitive mass spectrometer and has been further extended [12]. Analysis was performed with a QTrap 5500 LC-MS/MS System (Applied Biosystems, Foster City, CA, USA) equipped with TurboIonSpray electrospray ionization (ESI) source and a 1290 Series HPLC System (Agilent, Waldbronn, Germany). Chromatographic separation was performed at 25 °C on a Gemini C18-column, 150 × 4.6 mm i.d., 5 μm particle size, equipped with a C18 4 × 3 mm i.d. security guard cartridge (Phenomenex, Torrance, CA, USA).

ESI-MS/MS was performed in the time-scheduled multiple reaction monitoring (MRM) mode both in positive and negative polarities in two separate chromatographic runs per sample by scanning two fragmentation reactions per analyte. The MRM detection window of each analyte was set to its expected retention time ±27 s and ±48 s in the positive and the negative modes, respectively. Confirmation of positive analyte identification was obtained by the acquisition of two MRMs per analyte (with the exception of moniliformin and 3-nitropropionic acid, that exhibited only one fragment ion). This yielded 4.0 identification points according to the European Union Commission decision 2002/657 [53]. In addition, the LC retention time and the intensity ratio of the two MRM transitions agreed with the related values of an authentic standard within 0.1 min and 30%, respectively.

Quantification was performed using external calibration based on serial dilution of a multi-analyte stock solution. Results were corrected by apparent recoveries that had been determined by spiking five different blank samples at two concentration levels.

### 4.3. Statistical Analysis

Each particular metabolite concentration was evaluated by analysis of variance (ANOVA) to determine any differences between the years of sampling. The data were Log10 transformed to create a normal distribution. When the analysis was statistically significant, the post hoc Tukey’s multiple comparison procedure was used for separation of the means. Statistical significance was judged at the level *p* ≤ 0.01. All the analyses were done using SigmaStat for Windows Version 2.03 (SPSS Inc., Chicago, IL, USA).

## 5. Conclusions

A broad range (up to 77) of fungal metabolites was present in natural grasses (Poaceae) during two different years, and co-occurrence was the rule. It is important to point out that the profile of common metabolites was shared during both years of sampling, and also that the occurrence of important metabolites is not an exceptional phenomenon but seems to be very common. Some metabolites present such as type A trichothecenes (T-2 and HT-2), zearalenone and derivates are of concern for ruminants. Partial degradation in the rumen does mean that they are less toxic to cattle than to other animals, but some of these degradation products can be more toxic than the original mycotoxin. There are some studies on synergistic effects and only a few of them include those metabolites that were the most prevalent in our study. So, it cannot be ruled out that low levels of several mycotoxins might be more problematic than high levels of an individual mycotoxin, due to their synergistic relationship.
